# Enhancing Clinical Translation of Cancer Using Nanoinformatics

**DOI:** 10.3390/cancers13102481

**Published:** 2021-05-19

**Authors:** Madjid Soltani, Farshad Moradi Kashkooli, Mohammad Souri, Samaneh Zare Harofte, Tina Harati, Atefeh Khadem, Mohammad Haeri Pour, Kaamran Raahemifar

**Affiliations:** 1 Department of Mechanical Engineering, K. N. Toosi University of Technology, Tehran 19967-15433, Iran; farshadmoradikashkooli@ymail.com (F.M.K.); souri1996m@gmail.com (M.S.); samanezare1375@gmail.com (S.Z.H.); Tinaharati@ymail.com (T.H.); Atefekhadem820@gmail.com (A.K.); mohammadhaeripour@gmail.com (M.H.P.); 2 Department of Electrical and Computer Engineering, Faculty of Engineering, University of Waterloo, Waterloo, ON N2L 3G1, Canada; 3 Faculty of Science, School of Optometry and Vision Science, University of Waterloo, Waterloo, ON N2L 3G1, Canada; kraahemi@gmail.com; 4 Advanced Bioengineering Initiative Center, Multidisciplinary International Complex, K. N. Toosi Univesity of Technology, Tehran 14176-14411, Iran; 5 Centre for Biotechnology and Bioengineering (CBB), University of Waterloo, Waterloo, ON N2L 3G1, Canada; 6 Data Science and Artificial Intelligence Program, College of Information Sciences and Technology (IST), State College, Penn State University, Pennsylvania, PA 16801, USA; 7 Department of Chemical Engineering, Faculty of Engineering, University of Waterloo, 200 University Ave W, Waterloo, ON N2L 3G1, Canada

**Keywords:** drug delivery, nanomedicine, artificial intelligence, machine learning, deep learning, nanoinformatics, cancer, clinical translation

## Abstract

**Simple Summary:**

Two fields of artificial intelligence and nanomedicine are very effective tools in moving towards the goal of personalized medicine. Combination of these fields, *i.e.*, nanoinformatics, enables better access to patient data as well as better nanomaterials design. An ongoing challenge in all forms of drug administration for cancer patients is that drug synergy at any point in treatment is time-dependent, dose-dependent, and patient-specific. Moreover, high heterogeneities of intra-tumor and interpatient make it hard to rationally design diagnostic and dug delivery systems, as well as analyze their results. Integration of artificial intelligence methods (especially data mining, neural networks, and machine learning) can fill these gaps by using classification algorithms and pattern analysis to improve the accuracy of diagnosis, drug delivery, and treatment. In this study, the basic concepts in artificial intelligence are explained and the contributions of nanoinformatics in cancer treatment are reviewed.

**Abstract:**

Application of drugs in high doses has been required due to the limitations of no specificity, short circulation half-lives, as well as low bioavailability and solubility. Higher toxicity is the result of high dosage administration of drug molecules that increase the side effects of the drugs. Recently, nanomedicine, that is the utilization of nanotechnology in healthcare with clinical applications, has made many advancements in the areas of cancer diagnosis and therapy. To overcome the challenge of patient-specificity as well as time- and dose-dependency of drug administration, artificial intelligence (AI) can be significantly beneficial for optimization of nanomedicine and combinatorial nanotherapy. AI has become a tool for researchers to manage complicated and big data, ranging from achieving complementary results to routine statistical analyses. AI enhances the prediction precision of treatment impact in cancer patients and specify estimation outcomes. Application of AI in nanotechnology leads to a new field of study, *i.e.*, nanoinformatics. Besides, AI can be coupled with nanorobots, as an emerging technology, to develop targeted drug delivery systems. Furthermore, by the advancements in the nanomedicine field, AI-based combination therapy can facilitate the understanding of diagnosis and therapy of the cancer patients. The main objectives of this review are to discuss the current developments, possibilities, and future visions in naoinformatics, for providing more effective treatment for cancer patients.

## 1. Introduction

Nanomedicine is applying nanotechnology for healthcare, covers a wide range of clinical applications from diagnosis of various diseases such as cancer at one end of the spectrum, to the formulation of carriers for gene and drug delivery applications at the other spectrum of nanoscience in medicine. According to the development in nanotechnology, drug-loaded nanoparticles or nanocarriers have the potential to improve controlled release drug delivery systems (CRDDSs). CRDDSs reduce side effects to the surrounding tissues by delivering a drug to the tumor site [1,2,3,4]. Moreover, solid tumors have certain characteristics that make drug delivery to them very complicated and difficult. These characteristics, which are known as physical obstacles of drug transport in solid tumors, include elevated interstitial fluid pressure, dense extracellular matrix, hyper-permeable blood microvessels, and dysfunctional lymphatic system [5,6,7,8]. On the other hand, most current drug-loaded nanocarriers cannot be used for cargo transport and release, localized delivery, and tumor penetration due to the lack of capabilities for controllable navigation and self-propulsion. To address these limitations, several solutions are suggested, including manipulation the physicochemical properties of nanocarriers, multifunctional nanoconstructs, using internal/external field for drug release from nanoparticles, multi-stage drug delivery systems, application of micro-/nano-robots for drug delivery, to name a few [2,3,9,10,11,12]. Emerging micro-/nano-robots, as an appealing type of delivery carriers that can reduce systemic side effects of highly toxic drugs and improve the therapeutic efficacy, have been recently developed [13]. Nanorobotics, as a new area of nanotechnology, is about dealing with the cellular, molecular, or atomic structures of devices. Nanorobots can be designed for various applications such as the diagnosis and treatment of lethal diseases as well as identification of target molecules by their unique sensors [14,15].

The field of nanomedicine has significantly improved the diagnosis and therapy of many diseases such as cancer. For instance, imaging agents and nanoparticle-modified drug compounds have noticeably enhanced contrast efficiency and treatment outcomes. The emergence of AI presents an attainable opportunity for pharmaceutical application including drug discovery, drug delivery, and nanomedicine for cancer treatment [16,17,18,19,20]. AI can play an important role in developing nanomedicine-based treatment outcomes [1]. The integration of AI with nanotechnology (*i.e.*, nanoinformatics) leads to considerable improvements in drug delivery to solid tumors. Nanoinformatics developments and the use of machine learning (ML) and data mining, as a result of advancement in the nanotechnology field, has led to development of methods for predicting the structural and functional properties of nanoparticles. Data mining and ML can be utilized for prediction of biological properties of different nanoparticles related to their biomedical applications. These include the effect of particle physicochemical properties on cellular uptake, cytotoxicity, molecular loading, and molecular release considering manufacturing properties like nanoparticle size and polydispersity [21].

In this review paper, an overview of the methods of AI, which can improve the field of drug delivery to solid tumor and cancer nanomedicine, is proposed. The roles of AI in nano-sized drug delivery systems (*i.e.*, nanomedicine), integration of AI with nanorobots used for drug delivery, and AI-guided therapy in the clinical applications are the major areas that will be investigated in detail.

## 2. AI and Drug Delivery

Recently, the progress of novel systems for targeted drug delivery with minimum side effects and high efficiency has attracted increasing attention. In this regard, researchers have focused on controlled drug delivery in facing the challenges related to traditional drug delivery systems such as narrow therapeutic index, systemic toxicity, and controlling the doses of drugs in long-term therapy [22,23]. Utilization of the microfabrication technology to produce implantable microchips has a promising effect in controlled drug delivery [24]. Further, integrators, differentiators, intelligent control system, neural networks, and fuzzy logic have been involved in designing the control systems.

CRDDSs such as injectable, transdermal, implantable and oral systems present many advantages in comparison with conventional dosage forms. Improved patient compliance and diminished dosing frequency, reducing in vivo fluctuation of drug concentrations and keeping drug concentrations in an expected range, restricted side effects, and localized drug delivery are some of these advantages [25]. However, as a result of the sophistication of formulations, it is necessary to maintain the desired release rates; thus, CRDDS faces huge challenges during the developmental phase. Generally, during the development stage of these systems, the correlation between the process variables, formulation and characteristics are not well understood. Therefore, a statistical approach like response surface methodology (RSM) has been used for the formulation and development of CRDDS, but it has not performed effectively in the development of CRDDS due to some limitations. Artificial neural network (ANN) is a novel statistical approach for CRDDS development. It is beneficial when there is not a noticeable functional dependence between the inputs and outputs [26]. In addition, sophisticated biological data and nonlinear systems can be modeled by using ANNs. Solving the problems of multi-response and multi-variate systems, classifying cancer and predicting the secondary structures of proteins are other applications of ANNs [14,27]. The correlation between CRDDS drug release profiles as well as process factors and formulation is not implicit and linear. Hence, related networks can be utilized along with different types of ANN models. The association between the process variables and the formulation and response, like in vitro drug release profiles, can be represented by related ANN models. These networks, for example, can be utilized in in vitro drug release profiles of novel formulations. Response prediction can be performed by these formulations, which are formulated with various manufacturing and composition processes. Besides, the best possible process parameters and formulations can be selected by ANNs through the optimization of purposes. ANNs also have some applications in the CRDDS design. These applications, like the processes of manufacturing and optimization of formulations, are restricted but promising. A great number of them are useful in the field of oral CRDDS, such as in designing the pre-formulation stage of oral controlled release dosage forms. Moreover, optimization and prediction of various controlled release formulations like transdermal formulations and control released tablets, osmotic pumps, pellets, beads, microspheres, and nanoparticles can be conducted by ANNs [3,13,15].

### 2.1. AI Algorithms for Drug Delivery

The mathematical foundations of AI are used to describe the structure and parameters of various AI algorithms. More precise interpretation, management and analysis of complex functions or data are the result of using AI algorithms. In this regard, biological-based approaches (e.g., neural networks), probability theories, computational intelligence, and statistical pattern recognition methods are combined with AI. In drug delivery, predicting pharmacokinetics of novel therapeutics including their quantitative structure–activity relationship (QSAR) or quantitative structure–property relationship (QSPR), in vivo response, skin- or blood–brain barrier permeability, and proper dosing can be performed by AI tools. According to the significance of predicting the pharmacokinetic profiles of drug candidates, use of in silico tools may lead to cost reduction and increased efficiency. On this basis, ML techniques such as Gaussian process, support vector machine, k nearest neighbor, random forest, naïve Bayes classifier, regression tree and classification would be beneficial. ANNs can also be used for choosing the optimum gradient conditions in chromatography, designing the pre-formulations, analyzing the multi-variate nonlinear relationships in pharmaceutical area, predicting the drugs behavior, and creating nonlinear input–output mappings [14]. Table 1 presents a summary of some AI algorithms that are exploited in drug delivery research.

### 2.2. AI Applications for Drug Delivery

**Figure 1:**
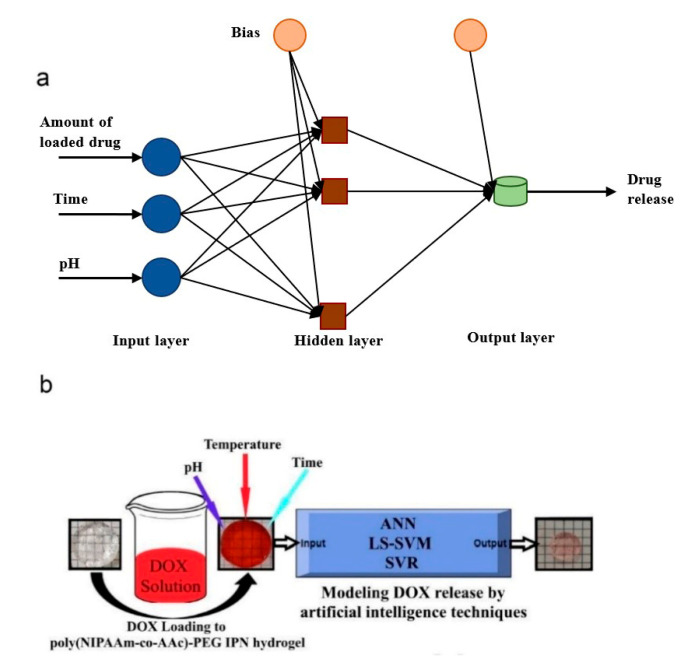
(**a**) Architecture of G-ANN in the analysis of release procedure. (**b**) Using ANNs, SVR and LS-SVM models for modeling the release behavior of DOX from temperature and pH responsive poly(NIPAAm-co-AAc)-PEG IPN hydrogel [34].

Some mechanisms of drug delivery systems may contain multiple steps and the release rate of each step is different. Furthermore, drug release is a kinetic procedure and the loaded drug may be affected by the amount of the released drug. Thus, to attain the appropriate release, the optimization of loading and release drug procedures in drug delivery systems should be conducted simultaneously. Besides, the functional dependence between the release and loading process is not intelligible. According to a study in 2018, integration of nonlinear generalized-artificial neural network (G-ANN) methods and experimental design has been proposed to optimize these processes concurrently. During the investigations, Curcumin and the functionalized PEGylated KIT-6 ([β-CD@PEGylated KIT-6]) nanoparticles have been determined as drug and nanocarrier, respectively. G-ANN has been exploited to optimize the curcumin release by inspecting the reaction of the loading step. The archived optimal parameters in the release procedure are 120 h of release time, 5.70 of pH and 1.80 of the weight ratios of drug to nanocarrier as well as 2.2 of weight ratio of drug/nanocarrier in the loading process and 43 h of loading time (Figure 1a). In spite of some methods like response surface methodology, there is no requirement of an explicit equation between the release amount and the factors in this method. This is the principal advantage of this approach [33].

In the procedure of drug release, prediction of the drug release kinetic of stimuli-responsive hydrogels with enough accuracy is very difficult. The reason is some environmental variables of the body like temperature and pH. However, modeling of the drug release behavior of these kinds of drug transporter materials has increasingly gained importance in industry and academic research. In a study reported by Boztepe et al., poly (NIPAAm-co-AAc)-PEG IPN hydrogels have been synthesized using free radical polymerization. These hydrogels have indicated rapid temperature- and pH-responsive deswelling behavior. Scanning electron microscopy (SEM) has been used for characterization of the surface morphology and textural properties of the hydrogels. It has shown that they have a porous microstructure. After loading the doxorubicin (DOX) to the hydrogels, increasing the release of this drug has been analyzed as a function of temperature, time and pH. The obtained experimental results have shown that the released amounts of DOX from the poly (NIPAAm-co-AAc)-PEG IPN systems are changeable depending on the mentioned factors. ANNs, support vector regression (SVR) and least squares support vector machine (LS–SVM) models have been applied to obtain the experimental DOX release data. As a result, ANNs have shown superior performance in modeling the complex and nonlinear release behavior of DOX from the IPN hydrogels. Therefore, ANN is an authentic method for studying the behavior of drug release from immensely swellable temperature and pH-responsive hydrogels (Figure 1b) [34].

Cell-penetrating peptides (CPPs) are a group of transporter systems that are often used to deliver different therapeutic agents into the cells [35]. These peptides can transport different types of particles and macromolecules into the cells [36]. For example, in the cancer research area, they can be exploited for tumor drug delivery. In this application, they can transport drugs to the deep regions of the tumor. According to a study, an innovative ML application has been developed to determine the interaction/insertion potential of CPPs into three different phospholipid monolayers. An ANN model has been developed, trained and tested after running tests on the experimental data. This neural network has accurately predicted the maximal change in surface pressure of different CPPs when administered below the membrane models. The ANN model can make a considerable depletion in cost and time. Thus, the insertion potential of different CPPs can be investigated before in vivo or experimental testing by using this tool. In conclusion, ANNs can pave the way for the process of designing the efficient gene and intracellular drug delivery systems [35].

Table 2 represents a summary of several studies, above mentioned and some other studies, exploiting AI for drug delivery. In addition to studies related to cancer, some target patients with other diseases like diabetes and obesity.

## 3. How AI Can Transform Nanomedicine

Currently, as cancer treatment methods are insufficient, advanced technologies are needed to detect, carry out drug delivery and treat cancer. The rapid growth of nanotechnology in nanomedicine is very promising for improving cancer treatment strategies. Nanomedicine promotes sophisticated targeting strategies and optimizes the effectiveness of existing anticancer compounds. Nanomedicine improves drug delivery, thereby reducing the side effects of anticancer drugs while increasing their effectiveness [42,43,44].

Synergistic drugs can be facilitated by optimizing drug combinations to improve the clinical efficacy of cancer therapy. However, optimization is complex because it entails selecting the right combination of drugs, dosage, and dose frequency to boost effectiveness and reduce unexpected toxic effects. Moreover, these effects may occur by the combination of drugs due to the complexity of biological systems. Therefore, combining AI and cancer nanomedicine can overcome the above challenges and increase the efficacy of cancer treatment [21].

AI methods have emerged in response to the need for unsupervised classifiers and predictors. Therefore, AI methods are widely used in nanomedicine, usually for accurate prediction. Due to the continuous growth of nanomedicine, AI methods can be applied to develop approaches to predicting the structural and functional properties and then optimizing therapeutic methods. The methods sought to predict the various properties, size, adhesion, molecular release, molecular loading, cytotoxicity, and cellular uptake.

Although AI methods are in the spotlight, the limitations of this approach should not be overlooked. The AI-based methods are data-intensive, which is a severe limitation in medicine. Overfitting and related constraints should always be considered in generalization processes.

AI methods have been extensively developed on the nanotechnology level through the pharmaceutical industry. Design, classification, monitoring, diagnosis, process control, scheduling, planning, and generation of options are the application areas of AI methods in nanotechnology [45]. AI methods have applications in nanomaterials, nanophysics, and nanomedicine as some fields of nanotechnology [46].

**Figure 2:**
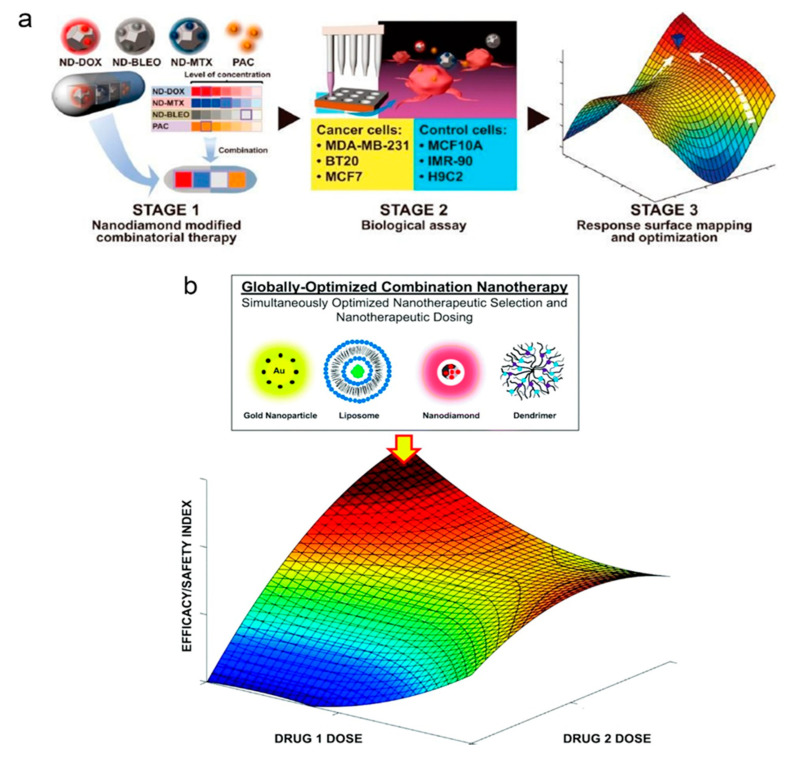
(**a**) The structure of feedback system control. Stage 1: Loading of bleomycin (BLEO), mitoxantrone (MTX), and DOX onto NDs was performed using physisorption, forming uniform and stable colloidal solutions, and combinations were designed. Stage 2: Using customized liquid handling robotic procedures, the drug combinations were applied to several types of cancers and control cells. The viability of cancer cell lines and control cell lines were utilized to feed into the informatics system. Stage 3: The informatics system provided cellular response surfaces by regression analysis with the customized statistic model on the combinations. Global combinatorial optimization was performed by differential evolution on the surface of the therapeutic window. Then the predicted randomized and optimum combinations were experimentally verified to confirm mapping accuracy [50]. (**b**) Make use of AI for nanomedicine optimization. A schematic doublet nanotherapy drug interaction map is present. Using rationally designed combination nanotherapy arrangements for initial calibration experiments [21].

Generally, the integration of nanotherapy and AI can be promising to improve personalized medicine. As a computational platform of AI, the quadratic phenotypic optimization platform (QPOP) identifies the effective drug combinations efficiently. The ratios and drug dose optimization in these combinations can be performed by this platform [47]. For example, in a study reported by Rashid et al., a QPOP has been developed to investigate an optimum combination from 114 FDA-approved drugs such as dactinomycin decitabine (Dec), mitomycin C (MitoC), and mechlorethamine. They want to treat bortezomib-resistant multiple myeloma according to this approach. The AI-guided optimization indicated that to treat this type of cancer, MitoC, Dec, and mechlorethamine was the superior three-drug combination. MitoC and Dec were the superior two-drug ones [48]. As an AI platform consisting of parabolic personalized dosing (PPD), CURATE AI helps select optimal doses given to patients during treatment. Moreover, using this platform, the risk of clinical execution decreases by circumventing the conventional design of clinical trials and translation of innovative nanotherapies and combinations [47]. In this regard, to treat metastatic castration-resistant prostate cancer, a CURATE.AI was utilized to guide the dosing of ZEN-3694 (a bromodomain inhibitor) and enzalutamide that were used in combination to treat metastatic castration-resistant prostate cancer to lessen serum prostate-specific antigen (PSA) levels. AI platform decreased prostate-specific antigen and stopped disease progression [49]. Therefore, the technologies are improved at every stage, from drug development to real-time in-human treatment by nanoparticles and carriers like nanodiamonds (NDs). Besides, the most effective integration of drugs and nanoparticles can be determined by some technologies like feedback system control (FSC) at every stage for use in nanotherapy [47]. For example, a feedback system control was developed to standardize drug dose combinations comprising one unmodified drug and three ND-modified drugs that would provide maximum cytotoxicity. These combinations were investigated on several breast cancer cell lines. The results represented a better performance of the nanomedicine drug combinations that were optimized using AI than an optimized unmodified drug combination and ND monotherapy or unmodified drug administration (Figure 2a) [50]. Although nanotechnology and nanotherapy have been beneficial in related areas, they are still critical before prevalent and usual clinic usage. ND-mediated therapy may be manifestly enhanced by the unique capabilities provided by AI [47].

The field of nanomedicine is also considering a group of adoptable carriers to enhance the localization of drug delivery and the targeting of disease sites. These carriers are helpful for combinatorial nanotherapy. In the future, they can increase the efficiency of treatments by simultaneous investigation of multiple disease pathways. Simultaneously, combination therapy could face some challenges such as enhanced targeting efficiency, rationally designed drug exposure, and preservation of drug synergy. The first promising step to promote treatment outcomes is reaching drug synergy. A combination therapy design presents multiple doses and drug parameter spaces that will be beneficial to optimize treatment globally. Furthermore, patients vary considerably regarding the dosages required to achieve drug synergy and the degree of drug exposure needed to achieve optimal treatment outcomes. Additionally, for the same patient, these parameters change over time. The appearance of AI can pave the way for accommodating this space into an actionable response for treatments. Combination therapy will be notably progressed with nanotechnology-modified therapeutics (Figure 2b) [21].

### 3.1. Clinical Treatment with AI

Nanomedicine is used in a variety of compounds in clinical cancer care. Being studied in clinical trials of patients with solid tumors, nanomedicine therapeutics include viral vectors, drug conjugates, lipid-based nanocarriers, polymer-based nanocarriers, and inorganic nanoparticles.

In addition to nanomedicine-based combination therapy design, AI would play a significant role in optimizing the administration of nanotechnology-modified as well as unmodified drug combinations. AI methods have been explored for clinical decisions to manage treatment in the clinic that have included big data-driven approaches, where electronic medical records of patient treatment outcomes, genetic and broader-omics profiling, and other information have been used for drug selection. These strategies collectively represent an important first step towards using valuable information databases to refine the regimen design process, which may improve broader patient population efficacy and safety. However, when regimens are selected, changing synergies and evolving patient responses to therapy still remain key challenges.

AI methods and computer vision have contributed to enhancing numerous aspects of human visual perception to detect meaningful clinical patterns. For example, imaging data have various applications, including segmentation of medical images, production, classification, and prediction of clinical datasets. Currently, AI methods are used in extensive academic research laboratories, technology companies, and biotechnology corporations in three main fields:

1. ML to predict the medicinal properties of molecular combinations and the targets of drug discovery [51];
2. Applying pattern segmentation and recognition techniques on medical images (including body surfaces and pathology slides, retinal scans, internal organs, and bones) for rapid diagnosis and tracking of the progression of diseases, as well as generative algorithms for computationally enhancing existing clinical and imaging datasets [52];
3. Development of AI methods in multimodal data sources, including clinical and genomic data, to identify novel predictive models [53].

**Figure 3:**
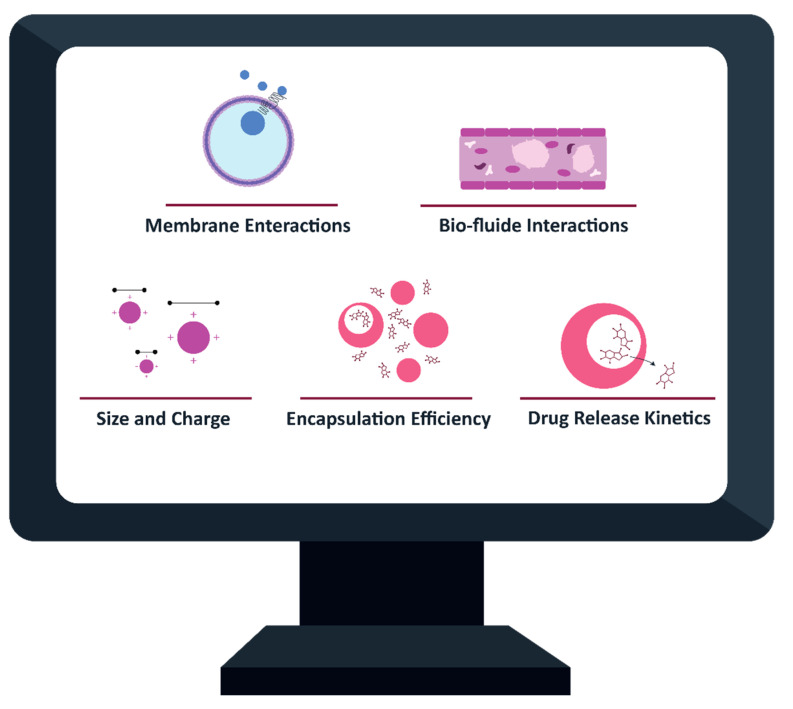
Computational methods promote different aspects of NP design. Available computational models and ML algorithms allow the prediction of NP charge and size, drug EE%, engaging with biomembranes, biofluids, and drug release kinetics; adapted from [55].

The use of AI in formulating nanotheranostics can provide an overview (Figure 3). To understand the molecular onset of cancer, nanomedicine enters the fray by providing many tools for the diagnosis and treatment of cancer. Nanomaterial-based delivery systems in theranostics (combination of diagnostics and therapy in one platform) provide better penetration of diagnostic and therapeutic agents into the body, which reduces the risk compared to conventional therapies. Predictive AI algorithms can be used to predict encapsulation efficiency (EE%) if there is a need to load imaging agents and drugs into the particle. For instance, a QSPR model was employed to determine if molecules are capable of being loaded into liposomes with more than 90% accuracy due to encapsulation process conditions and their chemical structure. Several algorithms were used to implement this model, such as decision tree, SVM, and iterative stochastic elimination (ISE). An identical approach was applied to predict the cytotoxicity of metal oxide NPs (MONPs). If this approach is also applied to other NPs, the effects of NP surface labeling for imaging on their biocompatibility can be investigated. Image analysis should also include the contribution of AI when medical imaging is discussed [54]. AI algorithms are improved for the detection and characterization, as well as continuous monitoring, of the reproducibility and accuracy of tumors to spare time and enhance the diagnostic capabilities of medical personnel. Our understanding of therapeutic efficacies and particle biological distribution profiles can be enhanced by implementing the above algorithms in imaging from nanotheranostics.

**Figure 4:**
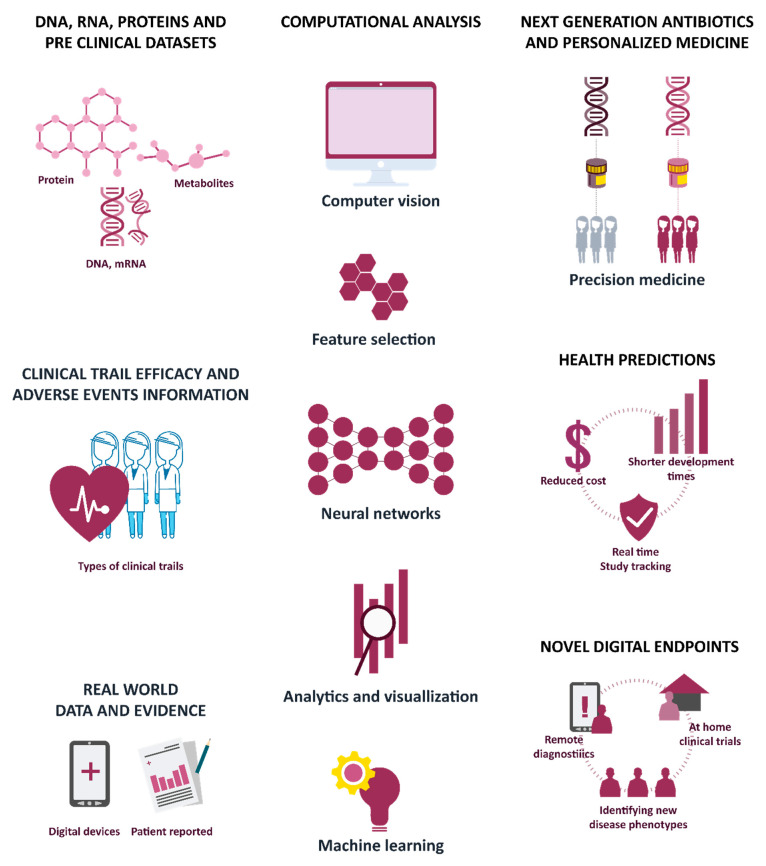
Applications of AI, computer vision, and ML in clinical development; adapted from [56].

The shared fields of computer science and medicine, the proactive regulatory perspective, and the accessibility of large datasets have examined applications, testing, and provide promising treatments to patients using advanced AI techniques (Figure 4).

Computation has a potentially significant effect on nanomedicine by improving the modeling and processing of information in nanomedicine [57]. Nanotechnological advances and computational resources make computation and informatics a key instrument to measure nanoscale toxicity. Nanoinformatics applications in nanomedicine include the analysis of nanoparticle-based pharmaceuticals for structure–activity relationships. Computational and theoretical *ab-initio* tools can deal with biomaterial nanosafety at such a scale since approximately all physicochemical properties, *e.g.*, concentration, shape, size, surface area, or electrostatic properties, can influence their interaction with the media nearby [58]. This offers a benefit in that it can be employed to create a targeted interaction with a certain biological medium; however, further research is still required to monitor nanotoxicity. New informatics instruments must be built and applied to effectively understand such interactions. Literature data may be utilized in modern computational techniques to demonstrate the connection between NP physical properties and biological interactions and, consequently, its toxicity [59]. Due to their small dimensions, certain concerns may be raised about their particular chemical, electrical, and optical properties. However, the mechanism of action (MOA) and dosage may lead to NP toxic therapy in certain situations [60]. A database of toxicity that is capable of being shared is needed to create successful nanoinformatics models. The Oregon Nanoscience and Micro-technologies Institute and National Institute for Occupational Safety and Health (NIOSH) are two examples of these databases [61]. Databases can be utilized to replicate the toxicity process and feed nanoinformatics models, thus minimizing the time spent translating NPs and drugs from the test phase to clinical use. Nanoscale data integration poses a range of challenges. These include the creation of NP toxicity databases and central repositories, information exchange and storage criteria, nano-oncology domain, and decision-making support tools [62].

### 3.2. Physicochemical Properties of Nanomedicine

Physicochemical properties of nanomedicine include cell uptake, cytotoxicity, molecular loading and molecular release. In addition to the properties mentioned, structural properties, such as nanoparticle size, adhesion and the polydispersity of nanoparticles determine the therapeutic activity of nanomedicines [63]. In this section, the effect of the application of machine learning and data mining on the prediction of nanomedicine properties is investigated.

#### 3.2.1. Cellular Uptake

Cellular uptake of nanoparticles overcome the plasma membrane because this membrane separates the space inside the cell from outside. It is important to know how these nanoparticles enter the cell because the intracellular fate and biological response depends on the proper uptake of nanoparticles. One of the first examples of machine learning and statistical modeling to predict the properties of nanomedicine by Puzyn et al. was a simple one-parameter linear regression model [64,65].

**Figure 5:**
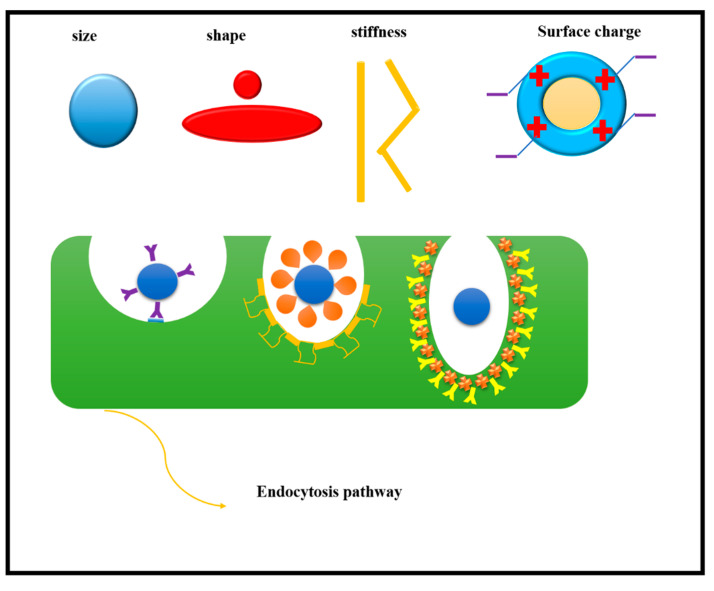
Cellular uptake with endocytosis pathways.

The use of nano-QSAR predictive models is very effective in cellular uptake because the cost of developing new nanoparticles is very high. QSRAs are a way to build computational mathematical models using the methods of GA, multivariate linear regression (MLR), and partial least squares regression (PLS). This model predicts properties through molecular descriptors and their coefficients [66]. Cellular uptake of nanoparticles occurs by a process known as endocytosis and is influenced by the physical and chemical properties of nanoparticles such as shape, size and chemical surface. As mentioned, one of the most important barriers to anticancer drugs is the inability to cross the plasma membrane, which is an effective barrier responsible for protecting living cells, severely restricts the entry and exit of macromolecular substances and may cause the drug to be blocked or restricted in a cell [64]. Nanoparticles are able to enter living cells through endostatic pathways, and also these nanoparticles enter directly into the cytoplasm, which is a good choice for drug delivery (Figure 5). The effect of endocytosis depends not only on the size of the nanoparticles but also on the amount of charge and surface coverage. Additionally, the adsorption properties determine the entry and exit of nanoparticles into the cell membrane. In order to improve cell uptake for targeted intracellular drug delivery, optimization of physical and chemical parameters is required [67]. Using nanoparticles of gold in different sizes, Wang et al. presented a report that used 34 nanoparticles and 29 types of descriptors. The k-nearest neighbors (k-NN) algorithm was also used in this model to improve cellular uptake in human lung and kidney cell cancers.

Due to the fact that a large number of parameters in QSAL model are already changed and the design space is very high, AI and data mining can be used. Winkler et al. studied cross-linked Iron Oxide (CLIO) nanoparticles using ML for cellular uptake. The data set contains 108 samples, which are divided into 28 samples for the test set and 78 samples for the training set. Linear and nonlinear QSAR models were used. The QSAR model was developed in line with multiple linear regression. The accuracy in this article reached 63, which was not a good accuracy. Several other articles show these results, which may have been due to the small size of the nanoparticles or the modification of the nanoparticles. Using a similar dataset has also caused this [67,68].

#### 3.2.2. Cytotoxicity

Nanoparticle cytotoxicity is defined as the extent to which the interaction of nanoparticles with cells disrupts cellular structures and/or processes essential for cell survival and proliferation. Cytotoxicity assays are a quick and simple way to perform initial acute toxicity assessments [69].

Cytotoxicity for healthy cells is a major challenge in drug delivery in the field of cancer treatment. For nanoparticles used to drug delivery, low toxicity and minimal environmental impact are prerequisite. One of the most successful and efficient methods for low toxicity is data mining. There are several parameters to determine the presence or absence of cytotoxicity in the laboratory. Many articles have discussed this, each exploring an issue, like different nanoparticles, different methods, and different parameters [70]. Nanoparticles can produce reactive oxygen species (ROS), which affects the concentration of intracellular calcium, activation of transcription factors, changes in cytotoxins as well as damage to DNA, interference with the cell pathway and changes in the process of gene transcription, etc. [71]. Oxidative stress can be a response to cell damage, considering oxidative stress caused by nanoparticles. It can have several causes: first—ROS can be used directly when both oxidants and free radicals are also present on the surface of the particles from the surface where nanoparticles are created [72]; second—by entering the mitochondria. Numerous studies have shown that very small nanoparticles can enter the mitochondria and physical damage that leads to oxidative stress can be created [73]; third—activation of inflammatory cells such as macrophages and neutrophils. Air sacs are involved in the process of nanoparticle phagocytosis. This can produce oxygen, and reactive species lead nitrogen [74]; fourth—metal nanoparticles (iron, copper, chromium, vanadium, etc.) can cause ROS production [75].

Horev-Azaria et al. predicted the degree of toxicity by classifying the available data as toxic and nontoxic, or the condition of cell survival and mouse lung incision using the decision tree method. The samples consisted of 151 samples and the decision tree consisted of two layers, and each layer had three offspring that included cell survival. It was observed that when cell survival was 30%, accuracy was 0.92, when cell survival was 25%, accuracy was 0.89, and when cell survival was 20%, accuracy was 0.85. This model showed the most important descriptor used as the nanoparticle concentration. The input conditions governing this test were cell type and contact time [76]. Winkler et al. used the Bayesian neural network (BNN) and 3200 data, which were divided into four categories according to biological conditions and different densities. The two linear and nonlinear models used in this study show that the nonlinear model performed better because the result reached an accuracy of 0.90. Finally, they found that apoptosis with CLIO nanoparticles depends on the type of coating and the surface [68]. Using descriptors and machine learning, Puzyn et al. were able to detect the cytotoxicity of 17 metal oxide nanoparticles [64].

#### 3.2.3. Molecular Loading

Molecule loading is an important property for nanoparticles. As mentioned, these particles have many uses, one of which is drug delivery or imaging, in which the loading of the molecule is very important. Generally, drug loading involves combining the drug in a polymeric matrix or capsule, i.e., the drug is released from the solid state and is prepared for absorption. Drug loading efficiency (DLE) and drug loading content (DLC) are two important factors for nanomedicines that DLC indicates, and DLE indicates the amount of drug used during the process. Loading capacity means dividing the total amount of trapped data by the total nanoparticle weight [77].

(1) $$Drug\;loading\;content\;\left(Weight\left[Wt\right]\%\right)=\frac{Mass\;of\;the\;drug\;in\;nanomedicines}{Initial\;mass\;of\;the\;nanomedicines}\times 100\%$$

(2) $$Drug\;loading\;efficiency\;\left(Weight\left[Wt\right]\%\right)=\frac{Mass\;of\;the\;drug\;in\;nanomedicines}{\;mass\;of\;the\;drug\;in\;feed}\times 100\%$$

In fact, DLC depends on the physical and chemical structure of the substance and DLE depends on the drug loading mechanism and the amount of drug in the feed and laboratory conditions. When the drug is loaded through physical absorption, the loading efficiency is poor, but when it is loaded through covalent bonding, high efficiency is observed. If the nanocarrier capacity is low, the loading process will not work properly. High loading is a prerequisite, but not a sufficient condition, for high drug loading efficiency. The drug loading content may have a greater impact on loading efficiency [78,79]. Winkler et al. used BNN to study the recovery rate of acetylcholinesterase (ACHE) bound to gold nanoparticles. The study consisted of 80 samples using 14 dragon descriptors that achieved 80% accuracy, but the researchers said the results were appropriate. Of course, with more accurate measurements and higher quality data, better results can be achieved [68]. Shalaby et al. used the ANN method in a study. In this study, the input variables were not accurately measured and were experimentally measured, which included the molecular weight of the polymer and the ratio of the polymer to the number of blocks. The accuracy was 91%, which was predicted for the noscapine trap. The results of using ML and data mining were very satisfactory, but with more data, better results are obtained [80].

#### 3.2.4. Nanoparticle Adhesion

Often, in the treatment and imaging of cancer and the advantage of extended permeability and retention of smaller nanoparticles, researchers limit the size of synthesized nanoparticles to 200–300 nm. This is not necessarily the best strategy for expanding the new therapeutic approach because there are many limitations to the treatment based on developed and penetrated therapy. Additionally, if nanoparticles are used to treat noncancerous diseases, they will not succeed on the basis of vascularization because the specificity of this method is to treat cancer. The nanoparticles are designed in studies to adhere to the walls of the patient’s blood vessels and prevent the excretion of hydrodynamic forces. Useful datasets are provided for data mining and ML to predict nanoparticle adhesion. Boso et al. used ANN to predict adhesion of fluorescent polystyrene nanoparticles to the vessel wall as a function of wall shear velocity and diameter of nanoparticles. This is important for developing an optimal structural formulation of nanoparticles to expand their density in diseased tissue [81]. Bozoyuk et al. were able to predict the size and potential of zeta in different conditions using artificial neural networks and machine learning. For cell adhesion by grouping the data, it was concluded that the nanoparticles that have more potential for zeta cause more adhesion [82].

#### 3.2.5. Polydispersity

One of the challenges and goals of the field of nanomedicine is the ability to produce finely dispersed nanoparticles. Nanoparticles typically exhibit relatively high disintegration, leading to several forms, such as nanoparticle mixing with changing loading capacity, reduced physical stability, variability of release profile and unpredictable degradation and clearance rate. Ismailzadeh-Gharehaghi et al. predicted the disintegration of chitosan nanoparticles using four input properties: amplitude of chitosan solution, chitosan solution sonication time, chitosan solution concentration, and pH of chitosan solution. The dataset used in this study contains 39 samples. The application of this model to the data evaluation reports R^2^ equal to 0.84. The data mining technique shows that when the concentration of chitosan solution increases, the disintegration decreases, and when the pH of the chitosan solution becomes more or less acidic, the disintegration rate increases [83]. Nanoparticles generally show a high degree of disintegration. A model with an artificial neural network was designed with polymer viscosity, contact angle and surface tension as input, and polydispersity as odor output. This model predicted a particle size between 7 and 400 nm. The particle prediction percentage was as follows: 2% for training, 4% for validation, and 6% for testing. Finally, it was found that the activity of the polymer surface has the greatest effect on particle size [84].

## 4. AI-Based Nanorobots for Drug Delivery

Nanorobotics is a new area of nanotechnology that deals with atomic, molecular, or cellular structure of devices. Nanorobots can be designed to diagnose and treat lethal diseases, and they have unique sensors for the identification of the target molecules. One of the most interesting research areas is the use of nanorobots to treat cancer. The primary reason for the advancement of nanorobotics is potentially cancer treatment. The progress of effectively targeted drug delivery to minimize the side effects of chemotherapy is an important aspect of ensuring effective treatment for patients. As drug carriers for prompt dosage regimens, nanorobots enable the maintenance, as required, of chemicals into the bloodstream for a longer period of time. Therefore, they provide the expected pharmacokinetic parameters for anticancer chemotherapy [13,15,85,86]. For molecular detection, diagnosis, and destruction in the initial phases of cancer progress of metastatic or malignant tumor cells, nanorobots often need a biosensing component. Nanorobots would lead to the development of personalized medicine. The advancement of nanotechnology has expanded the potential of nanorobots for screening and monitoring health conditions, and they can also be used for direct intravascular therapy. Nanorobots are also used to avoid aneurysm rupture and to cure it in critical cases. The intravascular capability of a nanorobot will decrease the amount of hemorrhaging by targeted drug delivery, and they can also be used to diagnose and treat cancer directly [87,88,89].

In chemotherapy, nanorobots will be used for treating cancer by precise chemistry dose. A similar approach could be taken to enable nanorobots to provide anti-HIV medicines. Medical nanorobotics are to be used in early diagnosis and targeted drug delivery for cancer treatment, tracking of diabetes pharmacokinetics and other health care methods. The use of injection nanorobots for cellular therapy in future medical nanotechnology is anticipated [90]. The advancement of micro/nano-electromechanical technologies has created the possibility of manufacturing implantable robots for a number of tasks, such as controlled delivery of drug organs. Due to significant developments in nanotechnology, the production of nanorobots, which are combined with external or internal power supplies, sensors and AI, has attracted increased interest. Nanorobots are quite promising for toxic and therapeutic detection. In the automation of molecular processing, the usage of AI offers the possibility to monitor nanorobots actions or movements. After intravenous injection, nanorobots are distributed into the bloodstream and their activity is promoted by bioactuation mechanisms. A new type of controlled drug release system is recommended for targeted therapy of a variety of disorders, particularly chronic disorders. The use of this system in personal medicine is extremely important. The successful nanodevices for drug delivery are magnetoelectric nanorobots. Magnetic fields monitor the position of these nanocarriers. The effectiveness and elimination of the adverse effects of the therapeutic agents are enhanced with magnetic resonance imaging (MRI) dependent on drug delivery systems including MRI propellants and monitoring devices, and control and nanocapsulates filled with medications. The usage of MRI-guided nanorobotics systems allows for the inactive targeting process of real-time control of nanocapsules. The use of ANNs for forecasting and improving the output of nanorobots incorporated with biosensors and transducers is a promising approach to tumor cell detection and selective drug delivery, which may have crucial importance in cancer therapy and decreased harmful drug reactions [14,91].

### 4.1. Use of Robots to Monitor Effectiveness of Treatment

In such cases as recovery, robotics can also be effective for assessing changes in human efficiency. One field where AI could be used helpfully is to track drug delivery to target organs, tissues or tumors. When the therapist tries to reach a tumor center which appears to be less vascular, anoxic but most active delivery problems arise. Researchers have sought to use a natural agent with the required properties as a supplement for “intelligent” nanoparticles to eliminate restrictions from mechanical or radioactive robotics. The external magnetic origin gives initial direction and such nanorobots can be linked covalently with therapeutic characteristics of nanoliposomes [14,91].

### 4.2. Intelligent System Design for Bionanorobots in Drug Delivery

Bionanorobots are the smart structures that are utilized for drug delivery, early diagnosis, and treatment at the cellular level. The damaging side effects of chemotherapy in cancer treatment lead to the use of bionanorobots in order to give the drugs directly to the tumor cells. In this way, the dose of the drug can be controlled, and the negative side effects can be reduced. Fluid shear stress is a significant factor in targeted drug delivery through nanorobots. In order to have precise modeling of tracking behavior and data transferring, some control tools such as AI, neural networks and fuzzy logic must be used. In the treatment of tumor cells, it is suggested to use a fuzzy logic-based intelligent system to decrease the false-positive rate in the diagnosis and increase controlled drug delivery [92]. In fact, the inspiration for in vivo applications of bio micro/nanorobots is to deliver precision drugs with greater speed to the target tumor. The distribution of fluorouracil medicines to suppress tumor growth in the model of the mice was magnetically driven by nanorobots. In order to deliver a significant amount of therapeutics in a site-specific of tumor, the drug release was activated externally [14,93].

## 5. AI Ability Compared to Other Methods

The advent of AI techniques can alter and improve the role of computers in engineering and science. In recent years, ML, as a subfield of AI, has evolved rapidly. The performance of statistical algorithms that are at the heart of ML applications enhances with training. Scientific models can be generated, tested, and refined using the growing infrastructure of machine-learning tools. Such methods are proper for addressing complicated problems. These problems may consist of nonlinear processes or combinatorial spaces where traditional procedures cannot find solutions or can deal with them just at a great computational cost [94]. ML-based algorithms can reduce a part of the computational burdens of simulations by partially replacing numerical methods with empiricism. The demand for massive training datasets is one of the major bottlenecks of ML algorithms. These datasets are provided by the terabytes of experimental and computational data gathered over the last few decades. Complex ML systems, like ANN, have the capability of highly nonlinear predicting with sufficient data. Extracting meaningful features of scientific value from the data is not always easy and can occasionally be as time-consuming as the experiments or computations providing them. Hence, sophisticated visualization techniques, signal processing algorithms, and statistics are essential for this task [95].

Finding a good balance between accuracy and computational resources is one of the key goals in simulations and computational modeling. ML methods can enhance the balance between speed and accuracy [95].

## 6. Challenging Issues and the Potential Solutions

Biomedical nanotechnology is devoted to investigating nanotechnology and nanoscience for health wellness, with the final aim of personalized health management. According to the reports of health agencies, technologies play an important role in cancer treatment, progression management, and monitoring. Additionally, it is proved that the diagnostics and treatment of a cancer can be more accessible, affordable, and sensitive by the introduction of nanotechnology assisted approaches. In biomedical research, nanosystems are beneficial for the development and design of therapies regarding patient profiles, which is personalized health management. Besides, notable features of numerical approaches, nano-assisted approaches such as AI including deep learning and machine learning, and bioinformatics can be very effective in the comprehension of predictions and trends [96].

Different problems like nanotechnology-related ones can be solved using AI tools. Additionally, data interpretation, complex tasks and computations, and drug design with lower side effects can be conducted using these powerful tools. Successful AI-based procedures result in development of functional, biocompatible, and stable drug delivery systems. In spite of advantages of AI methods, like continuous and fast performance of different tasks, they face several problems including high costs of maintenance, restoring, lost code recovery and system restoring, recurrent upgrading of software, the reflection of imprecision in the results, ethical concerns, risk of losing the data and lack of judgment, creativity, common sense or proper response to the altering environment. The mechanism of association between variables may not be clarified using ANNs models. Besides, achieving a reliable ANN-model requires a lot of time, application of sample size and big data for constructing more precise models. The reliability and performance of AI methods may negatively be affected by the deficiency of rational elucidation of the biological events. Some problems such as overtraining, overfitting, and the incorrect model validation can be overcome utilizing some methods like early stopping, Bayesian regularization, and training with dropout techniques.

ANNs are able to model complicated datasets and generate predictable models. However, with the concern of the clinical response to drug products, selecting proper datasets or algorithms in drug research attempts that are based on ANNs can be completely challenging. In this condition, the regulatory approval and commercialization of the products that are related with AI can be promoted by efficient annotation of datasets, application of appropriate techniques for more precise quantification of errors and uncertainties in the experimental procedures, model checkers for approving the performance of AI systems, computational methods for predicting the biological characteristics of molecules, optimizing algorithms to improve site-specific drug delivery and the computational efficiency, risk management, and personalized dosing [14].

AI algorithms also play a significant role in patient classification, optimizing properties of nanomedicines, and screening patients’ drug suitability. However, there are several challenges for the clinical implementation of these algorithms. Obtaining massive datasets for training the algorithms is one of the most essential issues to attain high accuracy in these computational methods. Thus, for the success of these models in clinics, data standardization along with data gathering from heterogenic populations of patients is pivotal. Moreover, a stronger relationship between the specialists in the fields of computer science, medicine, and nanomaterials and administration of computation in industrial and academic research will be helpful for clinical relevance and performance optimization [55,97].

## 7. Discussion and Concluding Remarks

Generally, AI technology has a massive impact on biomedical and biological sciences. The introduction of AI and related technologies in nanomedicine provides great hope for early detection of cancer and advanced cancer treatment in an effective way. AI and related technologies play a vital role in driving drug development in the last phase of clinical therapy. The use of AI overcomes the low response and failure of clinical trials due to inappropriate (below optimum) drug composition, thereby increasing the number of authorized drugs. Nanomedicine is used to increase drug targeting in specific situations to maintain drug coordination. However, the concentration of drug needed in the target position may vary from patient to patient. Additionally, drug coordination created with a particular dose for one patient may have adverse effects for another patient. It is believed that nanomedicine with AI has gained wide acceptance and has widened the gap between the information provided in the laboratory environment and the patient. The steady growth of the field of nanomedicine has led to the development of nanoinformatics and subsequently the use of data mining and ML to develop nano-QSARs and other methods to predict both functional and structural properties of nanoparticles. Research articles focusing on this area of research appear to be published in a wide variety of journals. The implementation of the predictors of dosing and the efficacy of treatment will aid nanomedicines to enhance their efficiency in clinical settings. Due to the continuous growth of nanomedicine, a development is witnessed in nanoinformatics and, thus, application of ML and data mining to develop approaches to predicting the structural and functional properties of NPs and then optimizing therapeutic methods.

Overfitting and related constraints should always be considered in generalization processes. ANN’s black-box nature is a constraint on ancillary health, where medics resort to quantitative methods to support their decisions although they cannot yet be replaced. Bayesian-regularized NNs helped determine the relationships between descriptors and response variables. They govern model complexity to strike a balance between variance and bias. In the first case, the model is very simple to record the basic relations between the data, and in the second case, the model is very complicated and fits the noise and the underlying bonds. An almost optimal method can be developed based on Bayesian regularization to regularize nonlinear NN regression models. The use of ML to integrate a variety of large-scale data provides a way for the prediction of the drug-neutralizing effects of the underlying molecular networks of the disease or leads to less toxicity. In this way, the best targets can be selected, and ultimately the efficacy can be predicted. Several ML-based approaches have been developed to identify the interactions between the target and the drug, critical for both the discovery of new drugs and the repositioning of drugs. The latest studies in the shared fields of computer science and medicine, the proactive regulatory perspective, and the accessibility of large datasets have examined applying, testing, and providing promising treatments to patients using advanced ML and AI techniques.

ML and AI grow exponentially and will soon become omnipresent. Two factors, *i.e.*, data accessibility and faster processing capacity, have contributed to the global upsurge of AI. There is an exponential growth in the volume of data produced since 90% of the data have been produced globally only over the previous two years.

## Figures and Tables

**Table 1:** AI algorithms that are used in drug delivery research and their applications.

Algorithm	Application in Drug Delivery	Mathematical Equation	Reference
Multilayer perceptron (MLP)	Predicting profile of drug dissolution, design of controlled release applications, optimization of the drug release profile and formulations	$y_{i}=f_{0}\left[\overset{N_{0}}{\underset{o=1}{\sum }}b_{o}+\overset{Nh}{\underset{h=1}{\sum }}W_{ho}\cdot f_{h}\left(b_{h}+\overset{Ni}{\underset{i=0}{\sum }}W_{ih}X_{i}\right)\right]$ *x_i_*, *y_i_*: the primary input and output, *fo*(·), *fh*(·): output and hidden functions, respectively, *W_ho_*, *W_ih_* (*i* = 1, 2, …, *Ni*, *o* = 1, 2, …, *No*): weights of connections between hidden and output units, and input and hidden units, respectively, *b_o_*, *b_h_*: biases of the output and hidden units	
Recurrent neural networks (RNNs)	Modeling or characterizing drug release from controlled release formulations	−	[28]
Artificial neural networks & Genetic algorithm (ANN&GA)	Optimization of the formulations such as controlled released ones, and optimization of the method of detection of similar biophenols in blood	−	[29,30]
General regression neural network (GRNN)	Dependable estimation of drug behavior in vivo and compensation of dissimilarities in the drug release kinetics under various conditions	$\hat{y}=\overset{n}{\underset{i=1}{\sum }}y_{i}exp\left(-D\left(x,x_{i}\right)\right)/\overset{n}{\underset{i=1}{\sum }}exp\left(-D\left(x,x_{i}\right)\right)\;$ $D\left(x,x_{i}\right)=\sum_{j=1}^{p}\left[xj-xi/\sigma_{j}\right]$^2^ *D*: distance between the point of prediction and training sample that is applied to elucidate the mechanism by which training samples show the prediction position (using *σ* as smoothness parameter)	[31,32]

**Table 2:** A summary of some studies utilizing AI methods for drug delivery.

Target Patients	AI Method	Study Focus	Year	Reference
Asthmatic patients taking monodisperse aerosols of salbutamol sulphate	ANNs	Estimating lung deposition, predicting aerosol behavior, and modeling the correlation between the in vitro data and in vivo effects	2010	[37]
Type 1 diabetic patients	ANNs	Identifying the glycemic regulation and patient dynamics	2012	[38]
Obese patients	Fuzzy logic models	Realizing the causes of obesity, averting obesity or diminishing its morbidity and mortality, and enhancing the quality of patient’s life	2012	[39]
Patients with colorectal cancer	An AI model	Determining the prerequisite for further surgery subsequent to the endoscopic resection of tumor and predicting the risk of lymph node metastasis	2018	[40]
−	G-ANNs	Optimizing the curcumin release by inspecting the reaction of the loading step	2018	[33]
Mellitus type 2 diabetic patients	ANNs	Designing the sustained-release matrix tablets carrying *Vaccinium myrtillus* leaf extract	2018	[41]
−	Machin learning (ML)	Determining the interaction/insertion potential of CPPs into three different phospholipid monolayers	2019	[35]
−	ANNs, SVR and LS-SVM models	Modeling the complex and nonlinear release behavior of DOX from the IPN hydrogels	2020	[34]

## Data Availability

The study did not report any data.
